# Dental Education

**Published:** 1867-07

**Authors:** 


### EDITORIAL DEPARTMENT.

---

*Dental Education.*—As the time approaches for the meeting of the American Dental Association, in Cincinnati, (on the last Tuesday in this month,) we are reminded of some of the views advanced at the last meeting held in Boston, by certain members of this body, during the discussion of the subject of Dental Education.

While the majority of those who engaged in this discussion did justice to the untiring devotion, both as regards energy, time and means, of the Professors of the different Dental Colleges; yet there were some who placed a very low estimate upon the labors of those, who have been instrumental in giving position to the profession of which they are members.

Dental Collegiate Education needs the support of the profession, and if the system is not altogether perfect in its theory, which we freely admit, how, we ask, is it to be improved by such Bœotian prejudices as it meets with? Every dental practitioner should be an advocate of Collegiate education and testify, by the pains he takes to encourage it, his sense of the treasure he or others have received. He should stand faithfully by those men who may meet with popular prejudice because of their efforts in behalf of a policy so wise; and should seize every opportunity by his pen, his lips and his influence, to lighten the burdens and responsibilities of those, upon whom such burdens fall. He should be an open and determined champion of all that tends to elevate the status of the profession; for by so doing, he will not only add to his own reputation, but will serve his vocation well, and guard the community from the arts of the empiric.

The demand for a high order of intelligence in our profession, was never more imperative than at the present, and the future promises to increase the demand almost beyond the power of human conception. Educate the members of a profession and you give rank and position to the profession itself.

